# Growth and development of syphilis-exposed and -unexposed uninfected children during their first 18 months of life in Suzhou, China: a nested case–control study with propensity score matching

**DOI:** 10.3389/fpubh.2023.1263324

**Published:** 2023-12-08

**Authors:** Tian Gong, Yi Zhong, Yaling Ding, Qianlan Wu, Mengxin Yao, Jieyun Yin, Yan Shao, Juning Liu

**Affiliations:** ^1^Suzhou Maternal and Child Healthcare Center, Suzhou Municipal Hospital, The Affiliated Suzhou Hospital of Nanjing Medical University, Suzhou, China; ^2^Jiangsu Key Laboratory of Preventive and Translational Medicine for Geriatric Diseases, School of Public Health, Medical College of Soochow University, Suzhou, China

**Keywords:** syphilis exposure, growth, development, infants, pediatric, Suzhou, China

## Abstract

**Background:**

With the successful implementation of Prevention of Mother-to-Child Transmission (PMTCT) policies, the proportion of infants with exposure to both syphilis and antibiotic medication *in utero* has increased in China, but there is limited evidence about the early growth and development of such infants.

**Methods:**

We conducted a retrospective nested case–control study based on data from the China PMTCT program conducted in Suzhou from 2016 to 2021. Propensity score matching (PSM) was employed to extract 826 syphilis-exposed but uninfected (SEU) infants and 1,652 syphilis-unexposed uninfected (SUU) infants from a total of 712,653 infants. Maternal characteristics were collected through questionnaires, such as parity, age, education level, smoking and drinking habits during pregnancy. Infantile characteristics were retrieved from medical records or via questionnaires, such as gestational age, gender, mode of delivery, Apgar scores, birth weight and length, outdoor time, vitamin D intake, and feed pattern. Mixed effects models, adjusting for potential influencing factors, were used to investigate the early infantile growth pattern of SEU and SUU infants. All statistical analysis were conducted using R (version 4.2.0).

**Results:**

Length and weight were slightly higher in SEU infants than in the SUU infants at some time points (months 0 and 18 for length, *p-*values <0.05; months 0, 6, and 18 for weight, *p* < 0.05). In the mixed effects model, SEU group was found to be associated with higher weight [exponentiated beta exp.(β) = 1.15, 95% Confidence Interval (CI) = 1.06, 1.25], length [exp(β) = 1.42, 95% CI = 1.14, 1.77], and BMI z-score [exp(β) = 1.09, 95% CI = 1.00, 1.19].

**Conclusion:**

With the effective prevention of congenital syphilis under the PMTCT program, SEU infants have non-inferior growth patterns during their first 18 months of life compared with SUU controls in Suzhou, China.

## Introduction

Developmental Origins of Health and Disease theory hypothesized that early infant growth and development have a profound influence on lifespan and health span (1). Syphilis is a systemic infectious disease caused by *Treponema pallidum*, which is mainly spread through sexual transmission and mother-to-child transmission (2). When left untreated, maternal syphilis would contribute to adverse pregnancy outcomes (including perinatal deaths and congenital syphilis) in 50–80% of infected pregnancies (3, 4). Furthermore, as the result of congenital syphilis, fetus and neonates may suffer from abdominal abnormalities (such as hepatomegaly, splenomegaly, and bowel abnormalities), fetal growth restriction, hepatosplenomegaly, bone damage, skin lesions and developmental delay (5).

Early detection and treatment (for example, penicillin) in pregnant women with syphilis is effective in preventing congenital syphilis (6, 7). In 2008, the World Health Organization (WHO) published a global guideline to reduce the incidence of congenital syphilis to less than 50 cases per 100,000 live births (8). In response to the call of WHO, China issued a series of Prevention of Mother-to-Child Transmission (PMTCT) policies in 2010 and successfully achieved WHO’s goals in 2016 (9–11). At the end of 2019, the reported incidence of congenital syphilis in China decreased to 11.87 cases per 100,000 live births, only one-sixth of the incidence in 2013 (12). By 2022, the incidence of congenital syphilis in Suzhou has been reduced to 2.53 per 100,000 live births (data not reported). In Zhejiang province in China, a neighboring province to Suzhou, the incidence of congenital syphilis in 2020 was reported to be 2.36 per 100,000 live births (13). With the success of the PMTCT policies, the proportion of syphilis-exposed but uninfected (SEU) infants, whose mother suffered from syphilis and administrated antibiotic medication (mainly penicillin), has been increasing. For this growing population, it has become increasingly imperative to measure their long-term growth and health outcomes.

Currently, studies on the early growth and development of SEU infants are still far from adequate. The only relevant study was conducted by Luo et al. in Zhejiang province of China, which enrolled 333 pregnant women diagnosed with syphilis during 2014–2015 and matched with 333 uninfected women based on maternal age and expected date of delivery (14). After adjusting for maternal age and household income, they found that syphilis exposure is irrelevant to the early growth of children under 18 months. As mentioned in the limitation section of this paper, the influence of nutritional status and feeding patterns was not controlled due to insufficient data (14). Furthermore, both maternal syphilis infection and infantile growth are closely linked to factors like maternal body mass index (BMI), education level, and parity (15–17), which should also be considered when matching SEU and syphilis-unexposed uninfected (SUU) infants.

Therefore, our study, based on a PMTCT program conducted in Suzhou City of Jiangsu Province, sought to use propensity score matching (PSM) that considered multiple maternal variables to achieve the following research objectives: (1) to compare the growth patterns of SEU and SUU infants from birth to 18 months; (2) to use mixed-effects models to identify the latent maternal and infantile factors affecting the growth and development of SEU and SUU children during the first 18 months in the Suzhou region.

## Methods

### Study design and population

The current study was a retrospective nested case–control study based on data derived from the PMTCT program conducted in Suzhou from January 1, 2016 to December 31, 2021. As reported in our previous study (7), the PMTCT program offers free syphilis screening and treatment to all pregnant women at the initial prenatal medical reservation. Information on all the participants and follow-ups of their infants was recorded in the PMTCT system. Pregnant women were diagnosed with syphilis when the results in toluidine red unheated serum/syphilis rapid plasma reagin test and enzyme-linked immunosorbent assay/treponemal pallidum particle agglutination were positive. Pregnant women diagnosed with syphilis were registered in the PMTCT system and then treated according to Chinese guidelines (18): in brief, two courses of penicillin therapy were administered, whereas the second one was located in the late trimester of pregnancy with an interval of more than 14 days between the first one. And the same antenatal care, except for syphilis treatment, are provided for both infected and uninfected pregnancies. If a woman with syphilis infection does not receive adequate treatment during pregnancy, her newborn children will be screened for syphilis and prophylactically treated periodically until a diagnosis of syphilis is ruled out.

A total of 712,653 infants born in Suzhou between January 1, 2016 and December 31, 2021, were initially recruited in our study. Nine infants infected with congenital syphilis were firstly excluded, then the exclusion was gradually made for (1) missing maternal information, such as age, early pregnancy BMI, parity, and educational level; (2) women with multiple births; (3) infants with fewer than three physical examinations between 0 and 18 months. Thereafter, 826 SEU infants and 473,906 SUU infants met the inclusion criteria.

**Figure 1:**
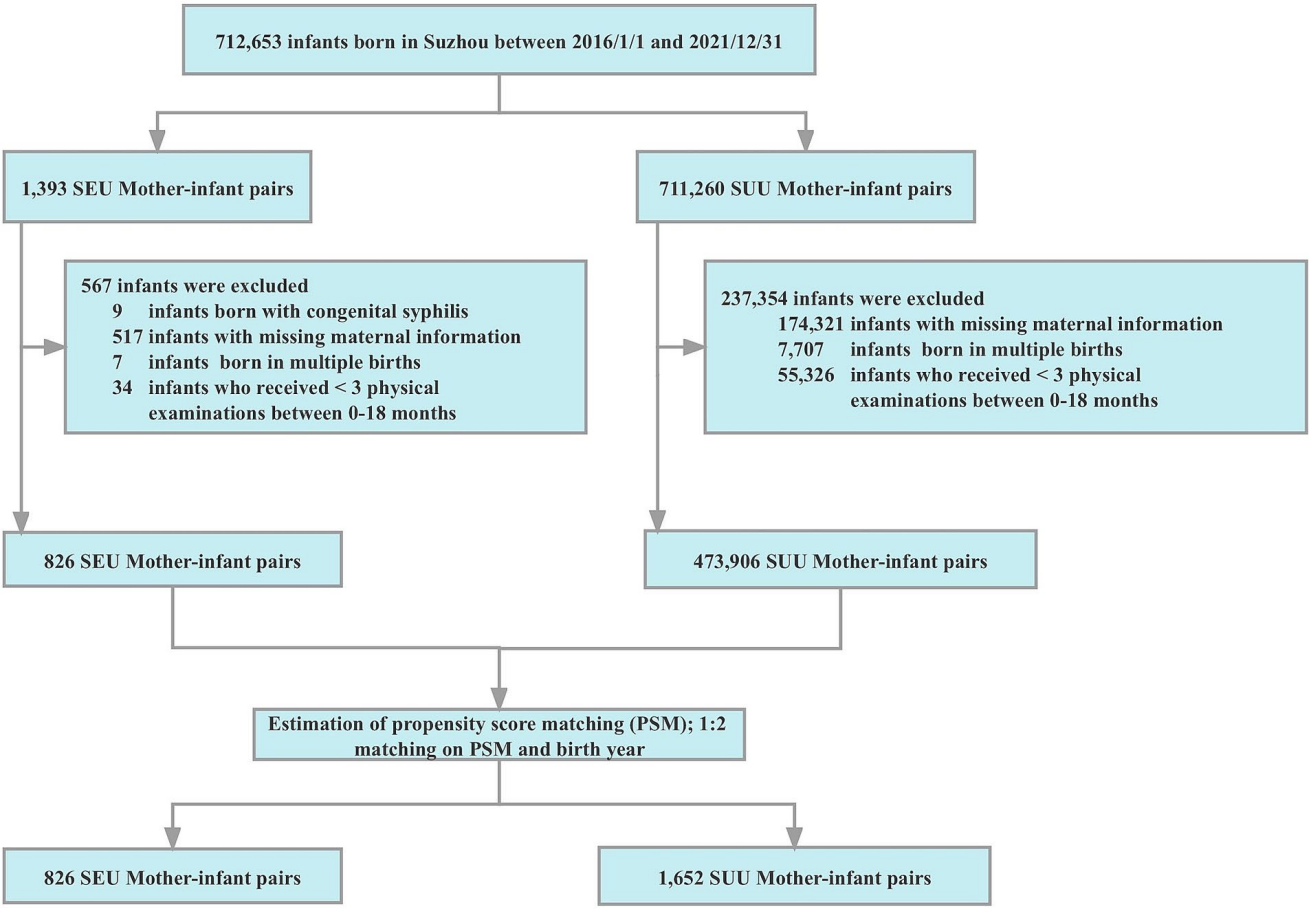
Flow chart for the selection and matching process of the study. PSM, propensity score matching; SEU, syphilis exposed uninfected; SUU, syphilis unexposed uninfected.

PSM is a statistical method that can reduce most of the bias caused by observed covariables to match for the most relevant factors (19). Therefore, we used PSM to minimize the imbalance in maternal characteristics between exposed and unexposed groups. The PSM was performed with nearest-neighbor matching of SEU and SUU infants born in the same year with a ratio of 1:2. Maternal factors (i.e., age, parity, education, early pregnancy BMI, and enrolled year) were considered as the matching variables. A caliper of 0.05 was set to ascertain the best match between the propensity score pairs. Ultimately, 826 SEU and 1,652 SUU mother-infant pairs were chosen. The selection and matching process of our study population is presented in Figure 1.

This study was approved by the ethics committee of the Suzhou Municipal Hospital (No. KL901417). Written informed consent was obtained from pregnant women, and relevant data of women and their infants were used in this study via an exemption.

### Infant and maternal anthropometric data

After exclusion for congenital syphilis, the follow-up for SEU infants is identical to that of SUU children in Suzhou. Both SUU and SEU children in the study had regular follow-up visits conducted by skilled professionals at months 0, 1, 3, 6, 8, 12, and 18 at the local maternal and child health hospital or community-based health service centers. Moreover, the child’s anthropometric data were measured and recorded every visit. Infant weight was measured to the nearest 0.1 kg with a digital scale in light clothing and no shoes. Infant length was measured to the nearest 0.1 cm with an infant-calibrated measuring mat. Infant BMI z-score was calculated by using the WHO Worldwide Child Growth guidelines (20). During the first 14 weeks of pregnancy, the mother’s weight was measured to the nearest 0.1 kg with a calibrated beam scale in light clothing and no shoes. The mother’s height was measured to the nearest 0.1 cm with a portable stadiometer. The mother’s BMI at early pregnancy was calculated as weight (kg) divided by the square of height (m). All anthropometric data were recorded based on the average of three measurements at one time point.

### Details of maternal/infantile characteristics

The demographic and obstetric characteristics of each pregnant woman, such as parity, age, education level, and smoking and drinking habits during pregnancy were collected through questionnaires. The infantile data, such as gestational age, gender, mode of delivery, Apgar scores at 1 and 5 min, birth weight and length, outdoor time, vitamin D (VD) intake, and feed pattern were retrieved from medical records or via questionnaires.

Maternal age was classified into four levels: <18, 18–24, 25–34, and ≥ 35 years. Early pregnancy BMI was divided into four levels: underweight (<18.5 kg/m^2^), normal (18.5–23.9 kg/m^2^), overweight (24.0–27.9 kg/m^2^), and obese (≥ 28.0 kg/m^2^) according to the China BMI classification standard (21). Education was separated into four levels: primary or less, junior school, high school, and college or above.

Children were separated into two groups, SEU or SUU infants, by the status of maternal syphilis infection. Premature birth was defined as delivery before 37 completed weeks of gestational age. For Apgar scores at 1 and 5 min after birth, scores <8 were considered abnormal. VD intake was divided into two levels (0–400 IU/d, ≥ 400 IU/d). Outdoor time was classified into two dosages (<2 h/day, ≥2 h/day).

### Statistical analysis

Statistical analyses were conducted using R (version 4.2.0). All reported *p-*values were two-sided, and the differences were considered statistically significant with *p* < 0.05. Continuous variables are summarized as means and standard deviation (SD), and categorical variables are expressed as numbers and percentages. Student’s t-tests and chi-square tests were employed to examine the differences of continuous variables and categorical variables between SEU and SUU children, respectively.

To effectively deal with repeated anthropometric measures data, we performed mixed effects models including potential confounders (22, 23). The growth curves in this study are shown in Equation 1, where i denotes each measurement in level 1 and j denotes pregnant women and infants in level 2.


(1) $$Y_{ij}=\beta_{0}+\beta_{1}\mathrm{Age}+\beta_{2}\mathrm{Group}+\beta_{3}X+\left(\mu_{0j}+\mu_{1j}\mathrm{Age}\right)+e_{0ij}$$

Sensitivity analysis was conducted by estimating the growth curves among SEU infants. The growth curves in this step are shown in Equation 2.


(2) $$Y_{ij}=\beta_{0}+\beta_{1}\mathrm{Age}+\beta_{2}X+\left(\mu_{0j}+\mu_{1j}\mathrm{Age}\right)+e_{0ij}$$

Weight, length, and BMI z-score were set as response variable $y_{ij}$ in turn. Group stands for the status of maternal syphilis infection. In addition to age and group, other potential maternal covariables (such as parity, age, drinking and smoking in pregnancy, education level, and early pregnancy BMI) and infantile covariables (such as sex, delivery way, feed pattern, VD intake and outdoor time) were put together into the models as confounding factors X. The variables $\mu_{0j}$ and $\mu_{1j}$ were random effects at the participant level (level 2), and e_0*ij*_ was the general random error at measurement level (level 1). Exponentiated coefficients and confidence intervals were calculated from the mixed effect models.

## Results

### Baseline maternal/infantile characteristics

Maternal demographic characteristics and clinical conditions before and after PSM are presented in Supplementary Table S1 and Table 1, respectively. Before PSM, SUU, and SEU infants had significant differences in maternal characteristics (including education and drinking habits during pregnancy) and infantile information (such as gestational age, birth weight, mode of delivery, VD intake, outdoor time, and exclusive breastfeeding). After applying PSM, the two groups became comparable with respect to education, drinking habits during pregnancy, VD intake, and early pregnancy BMI. The mean maternal age was 28.13 ± 4.41 years for SUU infants and 28.15 ± 4.43 years for SEU infants (*p* = 0.991). The mean gestational week at delivery was 38.93 ± 1.41 for SUU infants and 38.75 ± 1.47 for SEU infants (*p* = 0.003). Additionally, SUU infants tended to be delivered vaginally and exclusively breastfed in the first 6 months of life (all *p* < 0.001).

**Table 1:** Maternal and infantile characteristics between syphilis-unexposed and syphilis-exposed groups after matching.

Variables	Syphilis-unexposed	Syphilis-exposed	*p*-value
	*n* = 1,652	*n* = 826	
Maternal characteristics			
Age (years) (mean ± SD)	28.13 ± 4.41	28.15 ± 4.43	0.911
Education, n (%)			>0.999
Primary or less	144 (8.6)	72 (8.6)	
Junior school	747 (44.7)	374 (44.8)	
High school	386 (23.1)	193 (23.1)	
University or above	779 (46.6)	389 (46.6)	
Parity, n (%)			>0.999
Nulliparity	789 (47.2)	394 (47.2)	
Primiparity and multiparity	881 (52.8)	441 (52.8)	
Early pregnancy BMI, n (%)			>0.999
Underweight	155 (9.3)	78 (9.3)	
Normal	927 (55.5)	463 (55.4)	
Overweight	488 (29.2)	244 (29.2)	
Obese	100 (6.0)	50 (6.0)	
Smoking during pregnancy, n (%)	9 (0.8)	2 (0.3)	0.407
Drinking during pregnancy, n (%)	8 (0.7)	4 (0.7)	>0.999
Infantile characteristics			
Gestational week (mean ± SD)	38.93 ± 1.41	38.75 ± 1.47	**0.003**
Birth weight (kg) (mean ± SD)	3.34 ± 0.45	3.39 ± 0.43	**0.016**
Gender, n (%)			0.582
Female	469 (49.3)	401 (48.0)	
Male	847 (50.7)	431 (52.0)	
Mode of delivery, n (%)			**<0.001**
Cesarean	260 (36.8)	419 (50.2)	
Vaginal delivery	1,056 (63.2)	416 (49.8)	
Apgar at 1 min ≥ 8, n (%)	1,639 (98.7)	780 (98.4)	0.578
Apgar at 5 min ≥ 8, n (%)	1,658 (99.9)	789 (99.5)	0.173
VD intake ≥400 IU, n (%)	620 (50.7)	331 (51.6)	0.722
Outdoor time ≥ 2 h, n (%)	533 (43.2)	141 (22.5)	**<0.001**
Exclusive breastfeeding, n (%)	788 (59.3)	308 (44.4)	**<0.001**

SEU, syphilis exposed uninfected; SUU, syphilis unexposed uninfected; BMI, body mass index; VD, Vitamin D.The bold values means statistically significant with *p* < 0.05.

### Infant growth pattern and growth rate

**Figure 2:**
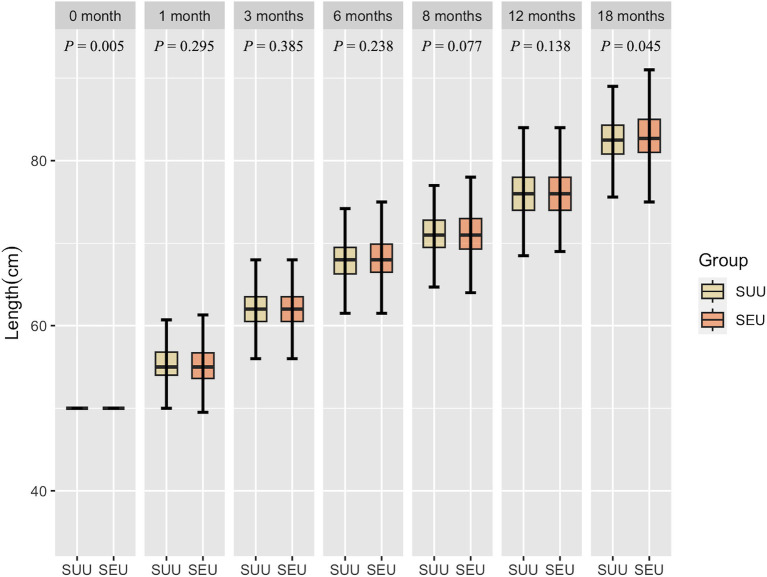
Boxplot of the length of children, grouped by syphilis exposure condition, at 0, 1, 3, 6, 8, 12, and 18 months after birth. SEU, syphilis exposed uninfected; SUU, syphilis unexposed uninfected.

**Figure 3:**
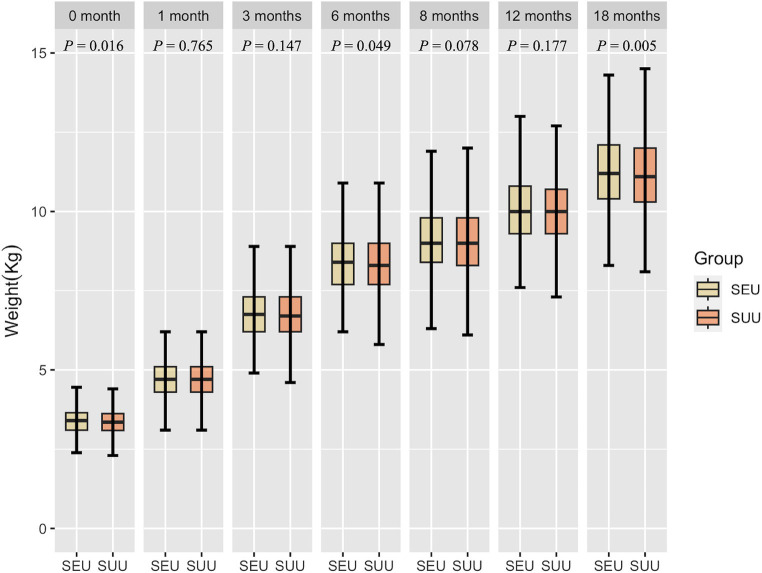
Boxplot of the weight of children, grouped by syphilis exposure condition, at 0, 1, 3, 6, 8, 12, and 18 months after birth. SEU, syphilis exposed uninfected; SUU, syphilis unexposed uninfected.

Figures 2, 3 show the growth patterns in length and weight of the infants in the two groups over 18 months. The growth patterns of SEU and SUU infants followed similar upward growth trends, but their growth rates slightly varied. The length of SEU infants was higher than that of SUU infants at 0 (*p* = 0.005) and 18 months (*p* = 0.045). The weight was higher in SEU infants at 0 (*p* = 0.016), 6 (*p* = 0.049), and 18 months (*p* = 0.005).

### Mixed effects models for growth indicators

Mixed effects models were fitted to explore the growth patterns of SEU and SUU infants during the first 18 months of life, as illustrated in Table 2. In our study, maternal syphilis exposure was found to be associated with higher weight {exponentiated beta [exp(β)] = 1.15, 95% Confidence Interval (CI) = 1.06, 1.25}, length [exp(β) = 1.42, 95% CI = 1.14, 1.77], and BMI z-score [exp(β) = 1.09, 95% CI = 1.00, 1.19]. The results also indicated that boys tend to have rapid change rates of weight [exp(β) = 1.55, 95% CI = 1.45, 1.68] and length [exp(β) = 3.74, 95% CI = 3.06, 4.57]. Collectively, sufficient consumption of VD and outdoor time were found to have positive impacts on infant weight, length, and BMI z-score, while early-pregnancy underweight and premature delivery exhibited a negative influence on these indexes. Primiparas, multiparous parity [exp(β) = 1.08, 95% CI = 1.00, 1.17], and exclusive breastfeeding [exp(β) = 1.08, 95% CI = 1.00, 1.17] were associated with a higher BMI z-score. Maternal overweight was associated with higher weight [exp(β) = 1.11, 95% CI = 1.01, 1.20] and BMI z-score [exp(β) = 1.15, 95% CI = 1.05, 1.26]. However, no significant effect was observed for other maternal factors, including age, education level, drinking, and smoking in pregnancy.

**Table 2:** Mixed effects models for weight, length, and BMI z-score in SUU infants and SEU infants.

Variables	Weight			Length			BMI z-score		
	Exponentiated beta	95%CI	*P*	Exponentiated beta	95%CI	*P*	Exponentiated beta	95%CI	*P*
Infantile characteristics									
Age (months)	1.45	(1.43,1.45)	**<0.001**	4.66	(4.62,4.76)	**<0.001**	0.99	(0.99,1.00)	**0.003**
Group	1.15	(1.06,1.25)	**<0.001**	1.42	(1.14,1.77)	**0.002**	1.09	(1.00,1.19)	**0.046**
Male	1.55	(1.45,1.68)	**<0.001**	3.74	(3.06,4.57)	**<0.001**	1.05	(0.98,1.14)	0.175
Vaginal delivery	1.03	(0.96,1.12)	0.401	1.40	(1.13,1.73)	**0.002**	0.95	(0.88,1.03)	0.231
Exclusive breastfeeding	1.07	(0.99,1.15)	0.078	0.86	(0.70,1.05)	0.143	1.08	(1.00,1.17)	0.050
Premature delivery	0.49	(0.41,0.59)	**<0.001**	0.16	(0.10,0.25)	**<0.001**	0.57	(0.48,0.68)	**<0.001**
Adequate VD intake	2.05	(1.93,2.18)	**<0.001**	7.46	(6.30,8.85)	**<0.001**	1.12	(1.06,1.17)	**<0.001**
Adequate outdoor time	1.77	(1.67,1.88)	**<0.001**	4.66	(3.97,5.53)	**<0.001**	1.07	(1.02,1.13)	**0.005**
Maternal characteristics									
Primiparas and multiparous parity	1.07	(0.99,1.15)	0.069	1.04	(0.85,1.28)	0.687	1.08	(1.00,1.17)	**0.042**
Age (years)	0.99	(0.98,1.00)	0.059	0.99	(0.97,1.01)	0.362	1.00	(0.99,1.00)	0.360
Drinking in pregnancy	1.39	(0.66,2.92)	0.383	2.69	(0.38,18.92)	0.320	1.38	(0.65,2.92)	0.399
Smoking in pregnancy	0.75	(0.35,1.62)	0.462	0.28	(0.04,2.10)	0.217	0.76	(0.35,1.65)	0.493
Education level									
Primary or less	0.90	(0.79,1.04)	0.161	0.73	(0.51,1.06)	0.097	0.99	(0.86,1.14)	0.921
Junior school	0.95	(0.86,1.04)	0.274	0.78	(0.60,1.02)	0.066	1.03	(0.93,1.14)	0.567
High school	0.92	(0.84,1.01)	0.097	0.84	(0.65,1.09)	0.200	0.93	(0.84,1.03)	0.185
University or above	Ref	Ref	–	Ref	Ref	–	Ref	Ref	–
Early pregnancy BMI									
Underweight	0.77	(0.67,0.89)	**<0.001**	0.54	(0.37,0.78)	**<0.001**	0.86	(0.74,0.99)	**0.033**
Normal	Ref	Ref	-	Ref	Ref	–	Ref	Ref	–
Overweight	1.11	(1.01,1.20)	**0.033**	1.07	(0.85,1.36)	0.541	1.15	(1.05,1.26)	**0.003**
Obese	1.09	(0.93,1.27)	0.264	1.27	(0.84,1.92)	0.262	1.13	(0.96,1.32)	0.139

CI, Confidence Interval; VD, Vitamin D; BMI, body mass index.The bold values means statistically significant with *p* < 0.05.

Supplementary Table S2 shows the result of the sensitive analysis among SEU infants. The influencing factors of SEU newborns were generally consistent with that of the whole population.

## Discussion

In this study, we investigate the growth pattern of SEU and SUU infants from birth to 18 months in Suzhou. In comparison to the SUU infants, SEU infants did not exhibit inferior growth patterns throughout their initial 18 months. Besides, the growth and development of infants were closely associated with maternal (including early pregnancy BMI) and infantile factors (including gender, premature birth, VD intake, and outdoor time).

Several studies have explored the growth and development of pathogen-exposed uninfected newborns. A Chinese retrospective cohort study found that infants born to HBV-positive pregnant women and healthy pregnant mothers were identical in early growth and development (24). Relevant studies of HIV-exposed uninfected (HEU) infants remain controversial. Some studies (25–28) suggested that HEU infants are more susceptible to physical growth retardation, which may be mainly attributed to socioeconomic factors (29, 30). In contrast, other studies (30, 31) suggested that the growth and body composition of HEU infants are not inferior to unexposed infants. According to Luo et al. (14), with universal coverage of therapeutic interventions for maternal syphilis, there was no negative connection between syphilis exposure in the uterus and early growth of infants under 18 months of age. In the current study, the incidence of congenital syphilis among fetal syphilis-exposed infants was only 0.2% (9 out of 1,393) during 2016–2021 in Suzhou region, which is similar to that of nearby regions (13, 14). Moreover, there were no remarkable differences in gestational age and birth weight between SEU and SUU infants in our study. These may be attributed to the successful implementation of PMTCT policies in Suzhou, which comprehensively detected local syphilis infections, raised public awareness of prevention, provided free treatment for pregnant women diagnosed with syphilis, and eventually reduced the rate of congenital syphilis. We also revealed that SEU infants have non-inferior growth and development to SUU controls before 18 months. SEU infants even have slightly higher weight and length at some time points (months 0, 6, and 18 for weight; months 0 and 18 for length). However, the statistical variations are not clinically meaningful, suggesting that fetal exposure to syphilis without infection does not appear to affect later growth parameters. The elevated growth parameter at birth may be induced by prenatal exposure to penicillin, which is the most suitable antibiotic treatment for syphilis (32). A study (33) in Shanghai, China revealed that penicillin used during pregnancy was associated with alterations in intrauterine growth and development of the fetus. Moreover, a systematic review reported that maternal penicillin intake may increase the risk of overweight and obesity in later childhood (34).

Our study provided insight into differences in prenatal and postnatal factors between SEU and SUU infants. Pregnant women with syphilis were more likely to deliver by cesarean section and to feed their babies with milk powder. The preferences in the delivery way and feeding pattern may affect later infantile growth and development. A modest association between exclusive breastfeeding and a higher BMI z-score was observed in our mixed-effects model. A systematic review study (35) found that, in well-developed regions, longer duration of breastfeeding was associated with slower growth rates in infants’ weight and height. However, another prospective cohort study suggested (36) that exclusive breastfeeding at <3 months may be associated with rapid growth in early childhood and body composition in young adulthood. Additionally, our study found that cesarean section may have a negative effect on the height growth pattern. A study from the United States supported our finding by concluding that infants delivered by cesarean section have lower length-for-age z-scores from birth to 12 months than infants delivered vaginally (37). Several studies (38–40) have identified a close relationship between the delivery way and the infantile gut microbiome. The bacterial communities of infants delivered via cesarean section were akin to those discovered on their mothers’ skin surfaces, while those of infants delivered vaginally were similar to those in their mothers’ vaginas (38). Thus, the bacterial communities that infants are exposed to at birth can significantly impact their long-term growth and development (41). Our study also found that primiparas and multiparous infants have higher BMI z-scores than nulliparas children. The potential reason may be because the physiological changes that occurred during pregnancy could create a more efficient foundation for the establishment and growth of subsequent pregnancies (42–44).

The growth and development of children are also closely linked to maternal weight status. Underweight and overweight at the early stage of pregnancy may be associated with lower and higher physical parameters, correspondingly. A meta-analysis showed that high maternal BMI compared with normal maternal BMI was associated with fetal overgrowth, while maternal underweight increased the risk of low birth weight in turn (45). It was suggested that the maternal weight represents the nutritional environment of the fetus. The fetus develops a specific adipose tissue memory system to adapt to the nutritional environment, which could profoundly affect its future nutritional status (46, 47).

Through mixed effects modeling analysis, boys were found to have higher physical parameters (weight, length, and BMI z-score) than girls. According to the WHO’s standard growth curve, boys have greater weight, length, and head circumferences than girls in their early ages (48). A Chinese multicenter prospective birth cohort study revealed similar results (49). Our study found that adequate VD intake and outdoor time can promote height and weight gain. Pérez-Fernandez et al. suggested that VD may stimulate growth hormone secretion and regulate gene expression in the human pituitary gland (50). Conversely, severe VD deficiency in children can lead to rickets, which is clinically manifested by poor body growth and skeletal deformities (51).

The present study has some strengths. First, the repeated measurements of the infant’s anthropometric data and the relatively large sample size could minimize errors caused by individual differences. Compared with the former study (14), the influence of maternal factors was fully considered and controlled through PSM. Additionally, given the scarcity of data on the growth pattern of SEU infants (14), our work could aid in understanding the impact of exposure to syphilis on growth in infancy.

## Study limitations

However, there are still several limitations that should be considered. Firstly, maternal and infant participants enrolled in this study were from Suzhou, a developed Southeast prefecture-level city in China, and thus may be geographically and ethnically constrained. Future studies should verify our findings in more regions and ethnicities and ultimately refine the growth pattern of SEU infants. Secondly, the current study was a retrospective study. Given the study’s observational nature, we were unable to derive a cause-effect relationship between these potential factors and infant growth. Thirdly, several factors were unavailable and uncontrolled, like maternal syphilis stage, whether to standardize treatment, months when specific antibody turns negative or to exclude syphilis infection, family socioeconomic status, marital status, and dietary habits, which further impeded us from more detailed subgroup analyses.

## Conclusion

Although SEU infants had slightly higher weights and lengths than SUU infants at some points in the first 18 months, the statistical differences were not clinically significant. The results indicated that with the effective prevention of congenital syphilis under the PMTCT program, SEU infants have non-inferior growth patterns during their first 18 months of life in Suzhou, China compared with SUU controls.

## Data availability statement

The raw data supporting the conclusions of this article will be made available by the authors, without undue reservation.

## Ethics statement

The studies involving humans were approved by the Review Committee of the Suzhou Maternal and Child Health Centre. The studies were conducted in accordance with the local legislation and institutional requirements. Written informed consent for participation was not required from the participants or the participants’ legal guardians/next of kin in accordance with the national legislation and institutional requirements.

## Author contributions

TG: Data curation, Writing – review & editing. YZ: Writing – original draft, Data curation, Software. YD: Writing – review & editing. QW: Writing – review & editing. MY: Software, Supervision, Writing – review & editing. JY: Writing – original draft, Writing – review & editing. YS: Supervision, Writing – review & editing. JL: Data curation, Investigation, Software, Writing – review & editing.
