# Supplementary material for: Growth and development of syphilis-exposed and -unexposed uninfected children during their first 18 months of life in Suzhou, China: a nested case–control study with propensity score matching
Source: Front Public Health. 2023 Dec 8;11:1263324. doi: 10.3389/fpubh.2023.1263324 (PMC10748380; doi:10.3389/fpubh.2023.1263324)
Supplement: Supplementary file 1 [file Table_1.docx]

Supplementary Material

**Table S1.** Maternal and infantile characteristics between syphilis-unexposed and Syphilis-exposed groups before matching.

| **Variables** | **SUU infants** | **SEU infants** | ***P-value*** |
| --- | --- | --- | --- |
|  | **n=473,906** | **n=826** |  |
| Maternal demographics |  |  |  |
| Age (years) (mean ± SD) | 28.01 ± 4.52 | 28.15 ± 4.43 | 0.352 |
| Education n(%) |  |  | **0.030** |
| Primary or less | 50,543 (10.7) | 72 (8.6) |  |
| Junior school | 101606 (21.4) | 181 (21.7) |  |
| High school | 92076 (19.4) | 193 (23.1) |  |
| University or above | 229,681 (48.5) | 389 (46.6) |  |
| Parity n(%) |  |  | 0.488 |
| Nulliparity | 217,671 (45.9) | 394(47.2) |  |
| Primiparity and multiparity | 256,253 (54.1) | 441 (52.8) |  |
| Early pregnancy BMI, n (%) |  |  | 0.101 |
| Underweight | 48,145 (10.2) | 78 (9.3) |  |
| Normal | 269,744 (56.9) | 463 (55.4) |  |
| Overweight | 135,471 (28.6) | 244 (29.2) |  |
| Obese | 20,546 (4.3) | 50 (6.0) |  |
| Smoking during pregnancy, n (%) | 581 (0.2) | 2 (0.3) | 0.569 |
| Drinking during pregnancy, n (%) | 584 (0.2) | 4 (0.7) | **0.009** |
|  |  |  |  |
| **Infantile characteristics** |  |  |  |
| Gestational week (mean ± SD) | 38.86 ± 1.39 | 38.75 ± 1.47 | **0.021** |
| Birth weight (kg) (mean± SD) | 3.34± 0.46 | 3.39 ± 0.43 | **0.010** |
| Gender, n (%) |  |  | 0.835 |
| Female | 227,266 (47.6) | 401 (48.0) |  |
| Male | 246,640 (52.4) | 434 (52.0) |  |
| Mode of delivery, n (%) |  |  | **<0.001** |
| Cesarean | 194,900 (41.1) | 419 (50.2) |  |
| Vaginal delivery | 279,006 (58.9) | 416 (49.8) |  |
| Apgar at 1 min ≥ 8, n (%) | 464,905 (98.9) | 780 (98.4) | 0.215 |
| Apgar at 5 mins ≥ 8, n (%) | 469,023 (99.8) | 789 (99.5) | 0.228 |
| VD intake ≥ 400IU, n (%) | 337,322 (89.4) | 595 (92.8) | **0.006** |
| Outdoor time ≥ 2h, n (%) | 331.598 (78.8) | 431 (61.4) | **<0.001** |
| Exclusive breastfeeding, n (%) | 249,407 (59.4) | 308 (44.4) | **<0.001** |
| Abbreviations: SEU, syphilis exposed uninfected; SUU, syphilis unexposed uninfected; BMI, body mass index; VD, Vitamin D. | | | |
|  | | | |

**Table S2.** Mixed effects models for weight, length, and BMI z-score in SEU infants.

| **Variables** | **Weight** | | |  | **Length** | | |  | **BMI z-score** | | |
| --- | --- | --- | --- | --- | --- | --- | --- | --- | --- | --- | --- |
|  | **Exponentiated beta** | **95%CI** | ***P*** |  | **Exponentiated beta** | **95%CI** | ***P*** |  | **Exponentiated beta** | **95%CI** | ***P*** |
| **Infantile characteristics** |  |  |  |  |  |  |  |  |  |  |  |
| Age (months) | 1.46 | (1.45,1.48) | **<0.001** |  | 4.76 | (4.66,4.85) | **<0.001** |  | 1 | (0.99,1.00) | 0.42 |
| Male | 1.65 | (1.45,1.88) | **<0.001** |  | 4.31 | (3.06,6.05) | **<0.001** |  | 1.09 | (0.95,1.26) | 0.207 |
| Vaginal delivery | 1.05 | (0.92,1.20) | 0.467 |  | 1.49 | (1.06,2.10) | **0.022** |  | 1 | (0.87,1.15) | 0.968 |
| Exclusive breastfeeding, n (%) | 1.08 | (0.95,1.23) | 0.252 |  | 0.95 | (0.68,1.34) | 0.776 |  | 1.12 | (0.97,1.28) | 0.124 |
| Premature delivery | 0.53 | (0.41,0.68) | **<0.001** |  | 0.17 | (0.09,0.33) | **<0.001** |  | 0.59 | (0.46,0.77) | **<0.001** |
| Adequate VD intake | 2.32 | (2.08,2.56) | **<0.001** |  | 9.58 | (7.1,13.07) | **<0.001** |  | 1.19 | (1.07,1.30) | **<0.001** |
| Adequate outdoor time | 1.63 | (1.48,1.80) | **<0.001** |  | 3.97 | (3.03,5.21) | **<0.001** |  | 1.02 | (0.95,1.09) | 0.592 |
| **Maternal Characteristics** |  |  |  |  |  |  |  |  |  |  |  |
| Primiparas and multiparous parity | 1.02 | (0.92,1.14) | 0.670 |  | 0.99 | (0.75,1.30) | 0.917 |  | 1.05 | (0.94,1.19) | 0.344 |
| Age (years) | 0.99 | (0.98,1.00) | 0.198 |  | 0.96 | (0.93,1.00) | **0.047** |  | 1.01 | (0.99,1.02) | 0.452 |
| Drinking in pregnancy | 0.89 | (0.35,2.27) | 0.802 |  | 0.91 | (0.07,11.36) | 0.944 |  | 1.19 | (0.44,3.16) | 0.741 |
| Smoking in pregnancy | 0.38 | (0.08,1.72) | 0.208 |  | 0.09 | (0,4.95) | 0.241 |  | 0.39 | (0.08,1.92) | 0.246 |
| Education |  |  |  |  |  |  |  |  |  |  |  |
| Primary or less | 0.91 | (0.72,1.15) | 0.441 |  | 0.68 | (0.37,1.25) | 0.21 |  | 0.98 | (0.76,1.25) | 0.847 |
| Junior school | 0.98 | (0.84,1.16) | 0.823 |  | 0.79 | (0.51,1.21) | 0.28 |  | 1.11 | (0.92,1.31) | 0.285 |
| High school | 0.90 | (0.76,1.05) | 0.174 |  | 0.67 | (0.43,1.03) | 0.071 |  | 0.98 | (0.83,1.17) | 0.851 |
| University or above | REF | REF | —— |  | REF | REF | —— |  | REF | REF | —— |
| Early pregnancy BMI |  |  |  |  |  |  |  |  |  |  |  |
| Underweight | 0.84 | (0.66,1.06) | 0.141 |  | 0.70 | (0.38,1.30) | 0.258 |  | 0.93 | (0.73,1.20) | 0.577 |
| Normal | REF | REF | —— |  | REF | REF | —— |  | REF | REF | —— |
| Overweight | 1.13 | (0.96,1.31) | 0.132 |  | 1.13 | (0.76,1.70) | 0.548 |  | 1.21 | (1.03,1.42) | **0.023** |
| Obese | 1.09 | (0.85,1.4) | 0.495 |  | 1.31 | (0.68,2.56) | 0.424 |  | 1.12 | (0.85,1.46) | 0.423 |
| Abbreviations: CI, Confidence Interval; VD, Vitamin D; BMI, body mass index. | | | | | | | | | | | |
